# Occupational exposure to petroleum-based and oxygenated solvents and hypopharyngeal and laryngeal cancer in France: the ICARE study

**DOI:** 10.1186/s12885-018-4324-7

**Published:** 2018-04-05

**Authors:** Christine Barul, Matthieu Carton, Loredana Radoï, Gwenn Menvielle, Corinne Pilorget, Simona Bara, Isabelle Stücker, Danièle Luce, ICARE study group, Anne-Valérie Guizard, Arlette Danzon, Anne-Sophie Woronoff, Michel Velten, Antoine Buemi, Émilie Marrer, Brigitte Trétarre, Marc Colonna, Patricia Delafosse, Paolo Bercelli, Florence Molinié, Simona Bara, Bénédicte Lapotre-Ledoux, Nicole Raverdy, Sylvie Cénée, Oumar Gaye, Florence Guida, Farida Lamkarkach, Loredana Radoï, Marie Sanchez, Isabelle Stücker, Matthieu Carton, Diane Cyr, Annie Schmaus, Joëlle Févotte, Corinne Pilorget, Gwenn Menvielle, Danièle Luce

**Affiliations:** 1 Univ Rennes, Inserm, EHESP, Irset (Institut de recherche en santé, environnement et travail)-UMR_S 1085, Pointe-à-Pitre, F-97110 France; 2 Univ Paris Sud, Paris Saclay University, Orsay, France; 3 Département de Biométrie, Institut Curie, DRCI, PSL Research University, Paris, France; 4 CESP, Cancer and Environment Team, INSERM U1018, Université Paris-Sud, Université Paris-Saclay, Villejuif, France; 5 Faculty of Dental Surgery, University Paris Descartes, Paris, France; 6 Sorbonne Universités, UPMC Univ Paris 06, INSERM, Institut Pierre Louis d’épidémiologie et de Santé Publique (IPLESP UMRS 1136), Paris, France; 7 The French Public Health Agency, Saint Maurice, France; 8 Ifsttar, UMRESTTE, UMR T_9405, Univ Lyon, Claude Bernard Lyon1 University, Lyon, France; 9 Manche Cancer Registry, Cotentin Hospital, Cherbourg-Octeville, France

**Keywords:** Solvents, Occupational exposure, Cancer, Larynx, Hypopharynx

## Abstract

**Background:**

To examine associations between occupational exposure to petroleum-based and oxygenated solvents and the risk of hypopharyngeal and laryngeal cancer.

**Methods:**

ICARE is a large, frequency-matched population-based case-control study conducted in France. Lifetime occupational history, tobacco smoking and alcohol consumption were collected. Analyses were restricted to men and included 383 cases of hypopharyngeal cancer, 454 cases of laryngeal cancer, and 2780 controls. Job–exposure matrices were used to assess exposure to five petroleum-based solvents (benzene; gasoline; white spirits; diesel, fuels and kerosene; special petroleum products) and to five oxygenated solvents (alcohols; ketones and esters; ethylene glycol; diethyl ether; tetrahydrofuran). Odds ratios (ORs) adjusted for smoking, alcohol drinking and other potential confounders and 95% confidence intervals (CI) were estimated with unconditional logistic models.

**Results:**

No significant association was found between hypopharyngeal or laryngeal cancer risk and exposure to the solvents under study. Non-significantly elevated risks of hypopharyngeal cancer were found in men exposed to high cumulative levels of white spirits (OR = 1.46; 95% CI: 0.88–2.43) and tetrahydrofuran (OR = 2.63; 95CI%: 0.55–12.65), with some indication of a dose-response relationship (p for trend: 0.09 and 0.07 respectively).

**Conclusion:**

This study provides weak evidence for an association between hypopharyngeal cancer and exposure to white spirits and tetrahydrofuran, and overall does not suggest a substantial role of exposure to petroleum-based or oxygenated solvents in hypopharyngeal or laryngeal cancer risk.

**Electronic supplementary material:**

The online version of this article (10.1186/s12885-018-4324-7) contains supplementary material, which is available to authorized users.

## Background

Despite a decrease in the last decades, incidence of hypopharyngeal cancer and laryngeal cancer in France among men remains among the highest in Europe, with annual incidence rates of about 5/100,000 and 7/100,000 respectively [1]. Tobacco smoking and alcohol consumption are the major risk factors [2], their joint effect being at least multiplicative [3].

Several occupational exposures are also known or suspected to be associated with these cancers. Exposure to strong acid mists [4] and to asbestos [5] are recognized risk factors for laryngeal cancer, and there is also some evidence that exposure to asbestos increases the risk of hypopharyngeal cancer [5]. Other possible occupational risk factors include exposure to polycyclic aromatic hydrocarbons, engine exhausts, and solvents [6]. Exposure to solvents in general was found to be associated with an increased risk of laryngeal or hypopharyngeal cancer in several studies [7–9], but the role of specific solvents was rarely investigated.

We previously examined the role of chlorinated solvents in head and neck cancer risk, and found an association between exposure to perchloroethylene and laryngeal cancer [10]. In our study population, increased risks of laryngeal and/or hypopharyngeal cancer were also observed among building caretakers, cleaners, farm workers, toolmakers, rubber and plastic workers, who may be exposed to other types of solvents [11]. Therefore, our objective in this study was to examine the associations between exposure to petroleum-based and oxygenated solvents and the risk of hypopharyngeal and laryngeal cancers.

## Methods

### Study design and population

The present study is based on data from the ICARE study, a French multicenter population-based case-control study, conducted between 2001 and 2007 in ten geographical areas covered by a cancer registry. Eligible cases were patients aged between 18 and 75 years, with histologically confirmed tumor of the oral cavity, pharynx, sinonasal cavities and larynx first diagnosed during the study period.

Population controls were selected in the same geographical areas using incidence density sampling, with frequency-matching by gender and age (< 40, 40–54, 55–64, ≥65 years old). A further stratification was performed to make controls comparable to the general population on socioeconomic status. The participation rates were 82.5% among cases and 80.6% among controls. Details about the study design have been described elsewhere [12]. The present study was restricted to squamous cell carcinomas of the larynx (International Classification of Diseases for Oncology 3rd revision codes: C32) and hypopharynx (C12-C13). Analyses were restricted to men and women were analyzed separately [13]. Overall, 383 cases of hypopharyngeal cancer and 454 cases of laryngeal cancer, and 2780 controls were included.

### Data collection

Standardized questionnaires were used by trained interviewers during face-to-face interviews to collect data. Those included sociodemographic characteristics, smoking and alcohol consumption histories, and a detailed lifetime occupational history, with a description of each job held for at least one month.

Trained coders blinded to case-control status coded occupations and industries, according to the International Standard Classification of Occupations (ISCO) [14] and the French Nomenclature of Activities (NAF) [15].

### Exposure assessment

Exposures to five petroleum-based solvents (benzene; gasoline; white spirits and other light aromatic mixtures; diesel, fuels and kerosene; special petroleum products) and five oxygenated solvents (alcohols; ketones and esters; ethylene glycol; diethyl ether; tetrahydrofuran) were assessed by job-exposures matrices (JEMs) developed for the French population in the context of the Matgéné program [16]. The JEMs assessed both inhalation and dermal exposures, with no distinction in exposure rating by route of exposure. Inhalation is, however, the main route of exposure for the solvents under study.

For each combination of ISCO and NAF codes, three indices of exposure were provided by the JEMs: (i) probability of exposure expressed as the percentage of exposed workers; (ii) intensity of exposure; and (iii) frequency of exposure as a percentage of working time. For these three indices, different categories were used according to the solvent (See Additional file 1). Exposure indices were provided for different calendar periods to take into account variations due to changes in exposure over time. Specific JEMs were also used to assess exposure to asbestos [17] and perchloroethylene [10]. Exposure to strong inorganic acids was obtained for each job from specific questions in the questionnaire.

Two variables were computed by linking lifetime occupational history with these indices: ‘ever/never’ exposed (‘ever’ defined as having worked in at least one job with probability of exposure greater than zero), and the Cumulative Exposure Index (CEI). CEIs were the results of summation over the entire work history of the product of exposure probability, frequency, intensity and duration of each job period, using the central value of the classes. CEIs were categorized in four categories: ‘never exposed’, and three categories according to the percentiles of the distribution among exposed controls (low: < 50th; medium: 50th–90th; high: > 90th). We estimated lifetime exposure prevalence to the various solvents as the mean of the maximum probability of exposure of each subject over his working life, using the central values of the classes.

### Other variables

Analyses were adjusted for age at interview in categories (< 40, 40–49, 50–59; 60–69, ≥70 years), residence area, alcohol consumption in categories (≤0.03, 0.04–2.00, 2.01–4.99, 5.00–7.99, 8.00–11.99, ≥12 glasses/day), smoking status (never; former: time since stopping smoking > 2 years at the interview; current), daily amount of tobacco in categories (1–10, 11–20, 20–25, > 25 g/day), duration of tobacco smoking in categories (1–20, 21–30, 31–40, > 40 years), and cumulative asbestos exposure in 4 categories (never exposed, and tertiles of the distribution among exposed controls).

### Statistical analysis

Adjusted odds ratios (ORs) and corresponding 95% confidence intervals (95% CI) were obtained by use of multivariable unconditional logistic regression models. Main analyses were performed separately for each solvent. Tests for linear trends were performed by modelling the median of each category as a continuous variable. We also examined the risks associated with joint exposure to several solvents by families of solvents (i) petroleum-based, (ii) oxygenated solvents. These analyses were restricted to combinations of solvents with at least 10 exposed cases.

In additional analyses we also adjusted for socioeconomic status, assessed by occupational class of the longest job held. As we previously found that exposure to perchloroethylene [10] was associated with laryngeal cancer, adjustment for this exposure was also performed but did not modify ORs estimates. Similarly, adjustment for self-reported exposure to strong inorganic acids did not change the ORs. Therefore, strong inorganic acids and perchloroethylene were not included in the models presented here.

## Results

Table 1 shows the main characteristics of cases and controls, as well as lifetime prevalence of exposure to the various solvents. Cases of hypopharyngeal and laryngeal cancers were generally older, more often blue collar workers, daily drank more alcohol and were more often smokers than controls. Exposure to petroleum-based solvents was relatively frequent, and exposure prevalences were higher in cases than in controls. Exposure prevalences were lower for oxygenated solvents, and were roughly similar in cases and in controls.

**Table 1:** Main characteristics of cases and controls

	Hypopharyngeal cancer *n* = 383		Laryngeal cancer *n* = 454		Controls *n* = 2780	
	n	%	n	%	n	%
Age						
< 40	0	0.0	5	1.10	76	2.7
40–49	51	13.3	56	12.3	555	20.0
50–59	164	42.8	175	38.6	825	29.7
60–69	118	30.8	147	32.4	939	33.8
≥ 70	50	13.1	71	15.6	385	13.9
Socioeconomic status						
Farmers	8	2.1	18	4.0	168	6.0
Self-employed workers	25	6.6	34	7.5	152	5.5
Managers	21	5.5	34	7.5	544	19.6
Intermediate occupations	32	8.4	55	12.2	564	20.3
Employees	40	10.4	45	10.0	297	10.7
Blue collar workers	252	65.8	265	58.8	1053	37.9
Alcohol consumption, glasses/day						
≤ 0.03	11	2.9	19	4.2	206	7.5
0.04–2.00	34	8.9	70	15.4	1190	42.8
2.01–4.99	89	23.2	110	24.2	849	30.5
5.00–7.99	78	20.4	108	23.8	305	10.9
8.00–11.99	82	21.4	74	16.3	134	4.8
≥ 12	70	18.3	54	11.9	73	2.6
Smoking status						
Never	3	0.8	13	2.9	753	27.1
Former	121	31.6	122	26.9	1271	45.7
Current	256	66.9	317	69.8	751	27.0
Lifetime exposure prevalence						
*Petroleum-based solvents*						
Benzene		16.9		15.6		11.7
Gasoline		11.6		11.1		8.7
Special petroleum-based products		3.6		2.6		2.5
Diesel, fuels and kerosene		20.0		18.4		14.4
White spirits		21.3		18.7		14.4
*Oxygenated solvents*						
Ketones and esters		13.5		11.8		9.5
Alcohols		12.2		11.5		12.9
Diethyl ether		0.4		0.6		1.4
Ethylene glycol		6.5		7.0		6.1
Tetrahydrofuran		1.6		1.0		0.7

The solvents studied are used as cleaners, degreasers and reagents in varied industrial processes, and are used in a number of formulations such as paints, adhesives, inks and dyes, dry cleaning solutions, pesticides, fuels, cosmetics and pharmaceuticals.

In our study population, men exposed to petroleum solvents were mainly employed as machinery fitters, and to a lesser extent as transport drivers (diesel/fuels/kerosene, gasoline) and construction workers (white spirits). Machinery fitters was the most frequent occupation among men exposed to ketones, esters, and ethylene glycol. Men exposed to ether and alcohols were mostly employed as medical workers. Exposure to tetrahydrofuran occurred primarily among plumbers and welders, due to the use of PVC pipe glues (data not shown).

Exposures to the solvents under study were correlated. The stronger correlations were found between exposures to benzene and white spirits (*r* = 0.62), gasoline and diesel/fuels/kerosene (*r* = 0.69) and ketones/esters and alcohols (*r* = 0.64) (see Additional file 2).

Table 2 reports the associations between occupational exposure to petroleum-based solvents and hypopharyngeal and laryngeal cancer risk. Overall, no significant association was found. For hypopharyngeal cancer, the ORs were slightly elevated for each solvent, on the order of 1.2, with no significant trend with cumulative exposure. The highest OR was found for the highest level of cumulative exposure to white spirits (OR = 1.46; 95% CI [0.88–2.43]), with some indication of a dose-response relationship (p for trend = 0.09). For laryngeal cancer, the ORs were around the null value of 1 for all solvents.

**Table 2:** Association between hypopharyngeal and laryngeal cancers and occupational exposure to petroleum-based solvents

	Controls	Hypopharynx			Larynx		
Petroleum based solvents	n	n	OR^a^	[95% CI]	n	OR^a^	[95% CI]
*Benzene*							
Never	2120	237	–		314	–	
Ever	552	102	1.07	[0.80–1.45]	109	0.94	[0.71–1.24]
CEI							
Low	279	51	1.15	[0.78–1.69]	46	0.81	[0.55–1.18]
Medium	220	39	1.02	[0.67–1.57]	56	1.27	[0.87–1.84]
High	53	12	0.9	[0.43–1.89]	7	0.42	[0.18–1.01]
p for trend				0.98			0.21
*Gasoline*							
Never	2161	243	–		323	–	
Ever	510	96	1.11	[0.83–1.50]	100	0.93	[0.70–1.23]
CEI							
Low	257	54	1.19	[0.82–1.72]	51	0.89	[0.62–1.27]
Medium	205	32	1.02	[0.65–1.61]	46	1.19	[0.80–1.77]
High	48	10	0.93	[0.42–2.06]	3	0.23	[0.07–0.79]
p for trend				0.87			0.03
*Special petroleum products*							
Never	2439	300	–		384	–	
Ever	234	38	1.20	[0.79–1.82]	39	0.93	[0.63–1.39]
CEI							
Low	118	18	1.22	[0.68–2.20]	17	0.86	[0.48–1.54]
Medium	93	17	1.21	[0.65–2.24]	20	1.07	[0.61–1.89]
High	23	3	1.10	[0.29–4.17]	2	0.64	[0.14–2.94]
p for trend				0.55			0.75
*Diesel, fuels and kerosene*							
Never	1753	175	–		239	–	
Ever	918	164	1.19	[0.90–1.56]	184	1.05	[0.82–1.35]
CEI							
Low	460	80	1.12	[0.80–1.56]	88	1.01	[0.74–1.37]
Medium	366	69	1.36	[0.96–1.93]	80	1.21	[0.88–1.67]
High	92	15	0.92	[0.48–1.75]	16	0.80	[0.43–1.48]
p for trend				0.61			0.41
*White spirits*							
Never	1436	125	–		186	–	
Ever	1240	216	1.14	[0.82–1.58]	237	0.93	[0.70–1.24]
CEI							
Low	620	93	1.15	[0.80–1.67]	112	0.99	[0.71–1.37]
Medium	494	86	0.99	[0.67–1.47]	94	0.84	[0.59–1.20]
High	126	37	1.46	[0.88–2.43]	31	0.97	[0.58–1.60]
p for trend				0.09			0.70

Abbreviations: *CEI* Cumulative Exposure Index

^a^OR adjusted for age at interview, residence area, alcohol consumption, smoking status, frequency and duration of smoking, exposure to asbestos

Regarding exposure to oxygenated solvents (Table 3), ever exposure to tetrahydrofuran was associated with non-significantly elevated risks of hypopharyngeal and laryngeal cancer. There was some evidence of a dose-response relationship for hypopharyngeal cancer, with a higher OR for the highest level of cumulative exposure (OR = 2.63; 95 CI% [0.55–12.65]; p for trend = 0.07). We also observed a significant increased OR for laryngeal cancer risk among men with the lowest level of exposure to ethylene glycol (OR = 1.75; 95% CI [1.04–2.94]) with a significant negative trend with the level of exposure (p for trend = 0.04). No association appeared between exposure to other oxygenated solvents and hypopharyngeal or laryngeal cancer risk.

**Table 3:** Association between hypopharyngeal and laryngeal cancers and occupational exposure to oxygenated solvents

	Controls	Hypopharynx			Larynx		
	n	n	OR^a^	[95% CI]	n	OR^a^	[95% CI]
*Ketones and esters*							
Never	2055	238	–		297	–	
Ever	618	100	1.01	[0.74–1.37]	126	1.10	[0.84–1.45]
CEI							
Low	309	46	1.05	[0.71–1.56]	62	1.24	[0.88–1.75]
Medium	245	38	0.89	[0.58–1.35]	53	1.08	[0.74–1.56]
High	64	16	1.34	[0.70–2.59]	11	0.87	[0.42–1.79]
p for trend				0.52			0.62
*Alcohols*							
Never	1775	225	–		290	–	
Ever	898	113	0.95	[0.72–1.27]	133	0.90	[0.70–1.17]
CEI							
Low	447	45	0.85	[0.59–1.25]	64	0.99	[0.71–1.37]
Medium	359	50	1.07	[0.74–1.55]	53	0.86	[0.60–1.22]
High	91	17	1.25	[0.68–2.31]	16	0.93	[0.51–1.72]
p for trend				0.31			0.87
*Diethyl ether*							
Never	2580	333	–		419	–	
Ever	90	5	0.59	[0.20–1.70]	4	0.37	[0.12–1.11]
CEI							
Low	45	1	0.28	[0.04–2.19]	1	0.2	[0.03–1.53]
Medium	36	4	1.41	[0.43–4.67]	3	0.73	[0.20–2.65]
High	9	0	–	–	0	–	–
p for trend				0.90			0.41
*Ethylene glycol*							
Never	2487	312	–		387	–	
Ever	183	26	0.82	[0.49–1.36]	36	1.00	[0.64–1.56]
CEI							
Low	92	15	1.08	[0.57–2.04]	28	1.75	[1.04–2.94]
Medium	72	11	0.83	[0.39–1.74]	6	0.39	[0.16–0.96]
High	19	0	–	–	2	0.34	[0.07–1.64]
p for trend				0.10			0.04
*Tetrahydrofuran*							
Never	2603	319	–		406	–	
Ever	67	19	1.67	[0.87–3.21]	17	1.39	[0.73–2.63]
CEI							
Low	35	7	1.33	[0.53–3.35]	9	1.50	[0.66–3.35]
Medium	26	8	1.57	[0.61–4.08]	8	1.62	[0.65–4.07]
High	6	4	2.63	[0.55–12.65]	0	–	–
p for trend				0.07			0.80

Abbreviations: *CEI* Cumulative Exposure Index

^a^OR adjusted for age at interview, residence area, alcohol consumption, smoking status, frequency and duration of smoking, exposure to asbestos

Further adjustment for socioeconomic status generally slightly decreased the ORs without any relevant change (see Additional file 3 for petroleum-based solvents and Additional file 4 for oxygenated solvents). Analyses considering exposure to combinations of petroleum solvents are shown in Table 4. Exclusive exposure to white spirits was associated with a non-statistically significantly elevated risk of hypopharyngeal cancer (OR = 1.40; 95% CI [0.85–2.32]), as well as other combinations including white spirits. Men exposed to the five petroleum solvents had an elevated risk of hypopharyngeal cancer (OR = 2.12; 95% CI [0.98–4.61]). An elevated risk of laryngeal cancer was found for exclusive exposure to diesel (OR = 1.62; 95% CI [0.89–2.96]), but not for exposure to diesel combined with other petroleum solvents. Combined exposure to benzene, special petroleum products and white spirits was associated with a decreased risk of laryngeal cancer (OR = 0.41; 95% CI [0.17–1.00]).

**Table 4:** Association between hypopharyngeal and laryngeal cancers and exposure to combinations of petroleum-based solvents

Exposure to petroleum-based solvents	Controls *n* = 2536	Hypopharynx *n* = 313		Larynx *n* = 396	
	n	n	OR^a^ [95% CI]	n	OR^a^ [95% CI]
None	1231	95	1	143	1
White spirits	316	46	1.40 [0.85–2.32]	55	0.98 [0.63–1.51]
Diesel	90	9	0.92 [0.41–2.11]	23	1.62 [0.89–2.96]
Ben, WS	74	19	1.55 [0.79–3.03]	26	1.27 [0.70–2.29]
WS, Diesel	242	44	1.47 [0.88–2.45]	46	0.90 [0.57–1.43]
Ben, SPP, WS	86	10	1.07 [0.47–2.45]	7	0.41 [0.17–1.00[
Gasoline, WS, Diesel	95	20	1.48 [0.75–2.90]	20	0.93 [0.50–1.73]
Ben, Gasoline, WS, Diesel	233	41	1.27 [0.75–2.15]	44	0.86 [0.54–1.39]
All petroleum-based solvents	55	15	2.12 [0.98–4.61]	14	1.28 [0.60–2.71]

Abbreviations: *WS* white spirits, *diesel* diesel, fuels and kerosene, *ben* benzene, *SPP* special petroleum products

^a^OR adjusted for age at interview, residence area, alcohol consumption, smoking status, frequency and duration of smoking, exposure to asbestos

The analysis of exposure to combinations of oxygenated solvents (Table 5) showed that men exposed to ketones, alcohols and tetrahydrofuran had a significantly higher risk of hypopharyngeal cancer (OR = 2.79; 95% CI [1.12–6.95]); an increased OR of borderline significance was also observed for laryngeal cancer (OR = 2.25; 95% CI [0.94–5.38]). No increased risks were found for other combinations including alcohols and ketones. No subject was exposed solely to tetrahydrofuran.

**Table 5:** Association between hypopharyngeal and laryngeal cancers and exposure to combinations of oxygenated solvents

Exposure to oxygenated solvents	Controls *n* = 2670	Hypopharynx *n* = 338		Larynx *n* = 423	
	n	n	OR^a^ [95% CI]	n	OR^a^ [95% CI]
None	1684	207	1	268	1
Ket	36	9	1.40 [0.57–3.48]	10	1.26 [0.55–2.92]
Alc, Ket	305	45	0.95 [0.63–1.43]	66	1.15 [0.81–1.63]
Alc, Etg	314	29	1.02 [0.63–1.65]	24	0.64 [0.40–1.05]
Alc, Ket, THF	25	10	2.79 [1.12–6.95]	10	2.25 [0.94–5.38]
Alc, Ket, Etg	158	23	0.84 [0.49–1.45]	27	0.86 [0.52–1.41]

Abbreviations: *OR* odds-ratio, *CI* confidence intervals, *Ket* ketones and esters, *Alc* alcohols, *Etg* ethylene glycol, *THF* tetrahydrofuran

^a^OR adjusted for age at interview, residence area, alcohol consumption, smoking status, frequency and duration of smoking, exposure to asbestos

## Discussion

In this study, we investigated the associations between occupational exposure to petroleum-based or oxygenated solvents and hypopharyngeal or laryngeal cancer. We did not find any significant excess risk when exposure to a single solvent was considered. Overall, our results do not suggest a substantial role of exposure to petroleum-based or oxygenated solvents in hypopharyngeal or laryngeal cancer risk.

Research on occupational exposure to solvents and the risk of hypopharyngeal and laryngeal cancers has been limited so far. In a case-control study conducted in Southern Europe, a significant increased risk of hypopharyngeal and laryngeal cancers was reported among men exposed to organic solvents [8]. In a multicenter case-control study in Central and Eastern Europe, exposure to organic solvents was associated with a non-significantly elevated risk of hypopharyngeal cancer, but no association was found with laryngeal cancer [9]. Other epidemiological studies which considered exposure to solvents in general did not report excess risks of laryngeal cancer [9, 18–21]. Most of these studies were case-control studies, that adjusted for smoking and alcohol [8, 9, 18–21], with the exception of a cohort study of construction workers, in which alcohol consumption was not available [21].

Exposure to solvents was assessed by industrial hygienists from detailed occupational histories in one study [9], with a JEM in three studies [8, 20, 21], and was self-reported in two studies [18, 19]. Very few studies have previously investigated exposures to specific petroleum-based and oxygenated solvents, so comparison of our results with the literature is limited.

As in our study, moderate and non-significant associations have been previously reported between laryngeal cancer and exposure to gasoline [9, 19] or to diesel, fuels and kerosene [9].

Our findings provide limited evidence of an increased risk of hypopharyngeal cancer in men exposed to white spirits, with elevated ORs for the highest level of cumulative exposure, and for men exposed to white spirits only or in combination with other solvents. Analogously, we previously reported non-significantly increased risks of hypopharyngeal and laryngeal cancer in women exposed to white spirits [13]. To our knowledge, only one study reported results on the relationship between exposure to mineral spirits and laryngeal cancer and found no association [9].

Our findings also suggest that exposure to tetrahydrofuran may increase the risk of hypopharyngeal cancer, and to a lesser extent the risk of laryngeal cancer. For hypopharyngeal cancer, the risk increased with cumulative exposure, and a significantly elevated OR was observed among men exposed to tetrahydrofuran, ketones and alcohols. The evidence is weaker for laryngeal cancer, with lower ORs and no indication of a dose-response relationship. Tetrahydrofuran has been recently classified by the International Agency for Research on Cancer as possibly carcinogenic to humans, with sufficient evidence of carcinogenicity in animals and no data in humans [22].

Overall, the associations between hypopharyngeal cancer and exposure to tetrahydrofuran and white spirits must be interpreted with caution and need to be replicated in other studies. Little information on possible underlying mechanisms is available. Exposure to tetrahydrofuran causes liver and renal tumors in rodents [22–24]. The mechanisms are not firmly established, but are probably different from that operating in the upper airways.

The evidence of carcinogenicity of white spirits in experimental animals remains limited [25]. Studies on the genotoxicity of tetrahydrofuran and white spirits are inconclusive [23–25] and mostly negative. The upper airways are nevertheless in direct contact with inhaled toxic agents. Respiratory tract irritation following inhalation exposure has been documented in humans and laboratory animals for both tetrahydrofuran [23, 24] and white spirits [25].

Chronic irritation, inflammation and increased cell proliferation is a possible mechanism, although there are no experimental data to support this hypothesis. Such mechanisms may be relevant to other parts of the upper respiratory tract and to the lower airways. It is worth noting that exposure to mineral spirits was previously found to be associated with squamous cell carcinomas of the lung [26] and oesophagus [27]. The association between exposure to tetrahydrofuran and cancer of the respiratory tract warrants further investigation.

Another possible mechanism is that eexposure to solvents may facilitate the penetration of carcinogens through the mucosa. To further investigate this hypothesis, we evaluate the presence of multiplicative interaction between smoking, alcohol drinking, asbestos exposure and exposure to white spirits and tetrahydrofuran. No significant interaction was found, but the statistical power was limited.

Our study has some limitations. We used JEMs to assess occupational exposures retrospectively. As JEMs do not take into account the heterogeneity of tasks within the same job title, they usually generate misclassification of exposure. On the other hand, JEMs assign exposure in a reproducible and automatic way, independently of the case or control status; consequently, misclassification of exposure is likely to be non-differential.

Non-differential misclassification of exposure leads to an average bias towards the null for dichotomous exposures, and tends to disrupt dose-response trends for multilevel exposure variables. Our positive findings are therefore unlikely to be explained by exposure misclassification [28].

We did not collect information about non-occupational solvent exposure, but the relative contribution of this source of exposure is likely to be minimal. Despite an overall large number of subjects, the prevalence of exposure to some solvents was low, resulting in large confidence intervals and limited ability for in-depth analyses.

Recall bias is possible, but was limited by the use of standardized questionnaires and the average number of reported jobs was similar in cases (4.2) and controls (4.6). Selection bias is probably not a major limitation of this study: controls had a distribution by socioeconomic status and lifetime prevalence of exposure to solvents comparable to that of the general population [16]; the distribution by age, sex and cancer site of the included cases was similar to that observed in France in the same period [29]. Finally, we assessed a large number of associations, and some findings may be due to chance.

Our study has important strengths. We used data from a large population-based case-control study, with sufficient statistical power to detect moderate associations. Availability of detailed information on lifelong occupational history allowed us to assess indices of cumulative exposure and to study dose-response relationships. We adjusted for smoking, alcohol consumption, the main non-occupational risk factors, as well as occupational exposure to asbestos. In additional analyses, we also took into account socioeconomic status and other occupational exposures, therefore residual confounding is likely to be minimal.

## Conclusions

This study provides weak evidence for an association between hypopharyngeal cancer and exposure to white spirits and tetrahydrofuran. Our findings do not suggest that the other petroleum-based or oxygenated solvents cause hypopharyngeal or laryngeal cancers.

## Additional files

Additional file 1: Categories of exposure indices. (PDF 140 kb)

Additional file 2: Spearman correlation coefficients between cumulative exposures to petroleum-based and oxygenated solvents. (PDF 193 kb)

Additional file 3: Association between hypopharyngeal and laryngeal cancers and exposure to petroleum-based solvents, with adjustment for socioeconomic status. (PDF 115 kb)

Additional file 4: Association between hypopharyngeal and laryngeal cancers and exposure to oxygenated solvents, with adjustment for socioeconomic status. (PDF 116 kb)

## Abbreviations

Alc: Alcohols
ben: Benzene
CEI: Cumulative Exposure Index
CI: Confidence interval
diesel: Diesel, fuels and kerosene
Etg: Ethylene glycol
Ket: Ketones and esters
OR: Odds-ratio
SPP: Special petroleum products
THF: Tetrahydrofuran
WS: White spirits
