# Supplementary material for: Occupational exposure to petroleum-based and oxygenated solvents and hypopharyngeal and laryngeal cancer in France: the ICARE study
Source: BMC Cancer. 2018 Apr 5;18:388. doi: 10.1186/s12885-018-4324-7 (PMC5887173; doi:10.1186/s12885-018-4324-7)
Supplement: Supplementary file 1 — Categories of exposure indices. (PDF 140 kb) [file 12885_2018_4324_MOESM1_ESM.pdf]

Categories of exposure indices

	Probability of exposure	Intensity of exposure	Frequency of exposure
**Gasoline**	<1%; 1–10%; 11–50%; 50–90%; >90%	not exposed; low; medium; high	<0.5%; 0.5–5%; 5–30%; 30–70%; >70%
**Special petroleum product**			
**Diesel, fuels and kerosene**			
**Benzene**		<0.1 ; 0.1–1 ; 1–5 ; 5–15 ; >15 ppm	
**White-spirits**		<1 ; 1–20 ; 20–50 ; >50 ppm	
**Ethylene glycol**	<1%; 1–10%; 11–20%; 21–30%;…; up to 91–100%	not exposed; low; medium; high	<1%; 1–10%; 11–20%; 21–30%;…; up to 91–100%
**Tetrahydrofuran**			
**Diethyl ether**		not exposed; very low; low; medium; high	
**Ketones and esters**			
**Alcohols**			
