# Supplementary material for: Occupational exposure to petroleum-based and oxygenated solvents and hypopharyngeal and laryngeal cancer in France: the ICARE study
Source: BMC Cancer. 2018 Apr 5;18:388. doi: 10.1186/s12885-018-4324-7 (PMC5887173; doi:10.1186/s12885-018-4324-7)
Supplement: Supplementary file 2 — Spearman correlation coefficients between cumulative exposures to petroleum-based and oxygenated solvents. (PDF 193 kb) [file 12885_2018_4324_MOESM2_ESM.pdf]

Spearman's correlation coefficients between cumulative exposures to petroleum-based and oxygenated solvents

	Ben	GAS	SPP	DIE	WS	KET	ALC	DIE	ETG	THF
BEN										
GAS	0.55									
SPP	0.42	0.09								
DFK	0.46	0.69	0.11							
WS	0.62	0.34	0.35	0.47						
KET	0.58	0.39	0.37	0.3	0.59					
ALC	0.37	0.19	0.29	0.1	0.32	0.64				
DIE	0.06	-0.06	0.21	-0.08	0.01	0.08	0.32			
ETG	0.52	0.58	-0.02	0.44	0.27	0.46	0.25	-0.05		
THF	0.05	-0.01	0.16	-0.04	0.16	0.29	0.06	0.05	-0.01	

Legend:
- < 0,10
- [0.10 ; 0.30 [
- [0.30 ; 0.60 [
- ≥ 0.60

Abbreviations: BEN= benzene; GAS = gasoline; SPP= special petroleum products; DFK = diesel,fuels and kerosene, WS= white spirits; KET= ketones and esters; ALC= alcohols; DIE = diethyl ether ; ETG= ethylene glycol; THF = tetrahydrofuran.
