# Supplementary material for: Occupational exposure to petroleum-based and oxygenated solvents and hypopharyngeal and laryngeal cancer in France: the ICARE study
Source: BMC Cancer. 2018 Apr 5;18:388. doi: 10.1186/s12885-018-4324-7 (PMC5887173; doi:10.1186/s12885-018-4324-7)
Supplement: Supplementary file 3 — Association between hypopharyngeal and laryngeal cancers and exposure to petroleum-based solvents, with adjustment for socioeconomic status. (PDF 115 kb) [file 12885_2018_4324_MOESM3_ESM.pdf]

Association between hypopharyngeal and laryngeal cancer and exposure to petroleum-based solvents, with additional adjustment for socioeconomic status

ORs adjusted for the same variables as in Table 2 + occupational class of the longest job held

	Controls	Hypopharynx			Larynx		
**Petroleum-based solvents**	**n**	**n**	**OR[a]**	**[95%CI]**	**n**	**OR[a]**	**[95%CI]**
Benzene							
Never	2120	237	-	-	314	-	-
Ever	552	102	0.92	[0.67 - 1.27]	109	0.86	[0.64 - 1.16]
CEI							
Low	279	51	0.96	[0.64 - 1.46]	46	0.73	[0.49 - 1.09]
Medium	220	39	0.89	[0.57 - 1.40]	56	1.17	[0.79 - 1.72]
High	53	12	0.80	[0.37 - 1.73]	7	0.43	[0.18 - 1.04]
p for trend				0.61			0.10
Gasoline							
Never	2161	243	-		323	-	
Ever	510	96	0.89	[0.65 - 1.23]	100	0.81	[0.60 - 1.09]
CEI							
Low	257	54	0.95	[0.63 - 1.41]	51	0.76	[0.52 - 1.12]
Medium	205	32	0.83	[0.51 - 1.34]	46	1.06	[0.70 - 1.61]
High	48	10	0.79	[0.35 - 1.81]	3	0.22	[0.06 - 0.77]
p for trend				0.58			0.01
Special petroleum products							
Never	2439	300	-	-	384	-	-
Ever	234	38	1.01	[0.64 - 1.58]	39	0.86	[0.57 - 1.31]
CEI							
Low	118	18	1.16	[0.61 - 2.22]	17	0.86	[0.47 - 1.59]
Medium	93	17	0.94	[0.49 - 1.80]	20	0.95	[0.52 - 1.72]
High	23	3	0.82	[0.21 - 3.16]	2	0.48	[0.10 - 2.27]
p for trend				0.94			0.49
Diesel, fuels and kerosene							
Never	1753	175	-	-	239	-	-
Ever	918	164	0.97	[0.72 - 1.31]	184	0.89	[0.68 - 1.17]
CEI							
Low	460	80	0.96	[0.67 - 1.38]	88	0.88	[0.64 - 1.23]
Medium	366	69	1.08	[0.73 - 1.59]	80	0.99	[0.69 - 1.40]
High	92	15	0.66	[0.33 - 1.29]	16	0.65	[0.34 - 1.29]
p for trend				0.37			0.25
White-spirits							
Never	1436	125	-	-	186	-	-
Ever	1240	216	0.99	[0.70 - 1.41]	237	0.83	[0.61 - 1.12]
CEI							
Low	620	93	1.11	[0.75 - 1.66]	112	0.94	[0.66 - 1.33]
Medium	494	86	0.77	[0.50 - 1.17]	94	0.68	[0.47 - 0.99]
High	126	37	1.23	[0.71 - 2.10]	31	0.85	[0.50 - 1.43]
p for trend				0.36			0.84
