# Supplementary material for: Occupational exposure to petroleum-based and oxygenated solvents and hypopharyngeal and laryngeal cancer in France: the ICARE study
Source: BMC Cancer. 2018 Apr 5;18:388. doi: 10.1186/s12885-018-4324-7 (PMC5887173; doi:10.1186/s12885-018-4324-7)
Supplement: Supplementary file 4 — Association between hypopharyngeal and laryngeal cancers and exposure to oxygenated solvents, with adjustment for socioeconomic status. (PDF 116 kb) [file 12885_2018_4324_MOESM4_ESM.pdf]

Association between hypopharyngeal and laryngeal cancer and exposure to oxygenated solvents, with additional adjustment for socioeconomic status

ORs adjusted for the same variables as in Table 3 + occupational class of the longest job held

	Controls	Hypopharynx			Larynx		
**Oxygenated solvents**	**n Co**	**n**	**OR[a]**	**[95%CI]**	**n**	**OR[a]**	**[95%CI]**
Ketones and esters							
Never	2055	238	-	-	297	-	-
Ever	618	100	0.9	[0.66 - 1.23]	126	1.02	[0.77 - 1.35]
CEI							
Low	309	46	0.97	[0.64 - 1.47]	62	1.13	[0.79 - 1.63]
Medium	245	38	0.75	[0.48 - 1.18]	53	0.97	[0.66 - 1.43]
High	64	16	1.12	[0.56 - 2.25]	11	0.77	[0.37 - 1.63]
p for trend				0.95			0.34
Alcohols							
Never	1775	225	-	-	290	-	-
Ever	898	113	1	[0.74 - 1.34]	133	0.94	[0.72 - 1.22]
CEI							
Low	447	45	0.93	[0.62 - 1.39]	64	1.05	[0.74 - 1.49]
Medium	359	50	1.01	[0.68 - 1.51]	53	0.85	[0.59 - 1.23]
High	91	17	1.17	[0.61 - 2.25]	16	0.92	[0.49 - 1.73]
p for trend				0.40			0.79
Diethyl ether							
Never	2580	333	-	-	419	-	-
Ever	90	5	0.77	[0.26 - 2.29]	4	0.45	[0.15 - 1.37]
CEI							
Low	45	1	0.17	[0.02 - 1.52]	1	0.15	[0.02 - 1.22]
Medium	36	4	2.01	[0.55 - 7.31]	3	1.00	[0.27 - 3.74]
High	9	0	-	-	0	-	
p for trend				0.56			0.55
Ethylene glycol							
Never	2487	312	-	-	387	-	-
Ever	183	26	0.80	[0.48 - 1.33]	36	0.98	[0.63 - 1.54]
CEI							
Low	92	15	1.02	[0.51 - 2.01]	28	1.71	[0.99 - 2.96]
Medium	72	11	0.89	[0.42 - 1.89]	6	0.41	[0.17 - 1.02]
High	19	0	-	-	2	0.36	[0.07 - 1.80]
p for trend				0.06			0.03
Tetrahydrofuran							
Never	2603	319	-	-	406	-	-
Ever	67	19	1.46	[0.76 - 2.83]	17	1.27	[0.67 - 2.42]
CEI							
Low	35	7	1.12	[0.42 - 3.01]	9	1.40	[0.58 - 3.37]
Medium	26	8	1.46	[0.52 - 4.09]	8	1.58	[0.60 - 4.16]
High	6	4	3.10	[0.61 - 15.71]	0	-	-
p for trend				0.13			0.63
